# Inhibitory Activity of *Boesenbergia rotunda* (L.) Mansf. Rhizome towards the Expression of Akt and NF-KappaB p65 in Acetic Acid-Induced Wistar Rats

**DOI:** 10.1155/2020/6940313

**Published:** 2020-05-20

**Authors:** Aziiz Mardanarian Rosdianto, Irma Melyani Puspitasari, Ronny Lesmana, Jutti Levita

**Affiliations:** ^1^Department of Pharmacology and Clinical Pharmacy, Faculty of Pharmacy, Universitas Padjadjaran, Sumedang 45363, Indonesia; ^2^Veterinary Medicine Program, Faculty of Medicine, Universitas Padjadjaran, Sumedang 45363, Indonesia; ^3^Center of Excellence in Higher Education for Pharmaceutical Care Innovation, Universitas Padjadjaran, Sumedang 45363, Indonesia; ^4^Physiology Molecular Laboratory, Central Laboratory, Universitas Padjadjaran, Sumedang 45363, Indonesia; ^5^Department of Medical Basic Sciences, Faculty of Medicine, Universitas Padjadjaran, Sumedang 45363, Indonesia

## Abstract

**Materials and Methods:**

Forty-eight male Wistar rats were divided into anti-inflammatory mechanism study (*n* = 18) and acute toxicity study (*n* = 30). The anti-inflammatory mechanism study employed six groups (*n* = 3), e.g., the normal control, negative control, positive control (quercetin 20 mg/kg BW), and three doses of BREE (250 mg/kg BW; 500 mg/kg BW; 1000 mg/kg BW). All groups (except the normal control) were inflammatory-induced *i.p.* using 0.1 mL of 1% of acetic acid. The expression of Akt and NF-kappaB p65 in the stomach and intestine of the rats was examined using Western blot analysis. The acute toxicity study (21 days) was conducted by following the Regulation of Indonesia National Agency of Drug and Food Control No. 7/2014 about In Vivo Nonclinical Toxicity Study using 5 doses of BREE (250 mg/kg BW; 500 mg/kg BW; 1000 mg/kg BW; 2000 mg/kg BW; 4000 mg/kg BW).

**Results:**

BREE reduces the infiltration of inflammatory cells in both the stomach and the intestine of acetic acid-induced rats. BREE also alters the expression of Akt and NF-kappaB p65 in the rat's stomach and intestine (*p*=0.005). The acute toxicity study reveals no lethal effects and behavioral signs of toxicity at all tested doses, which indicates that the LD_50_ is greater than 4000 mg/kg BW.

**Conclusion:**

Taken together, BREE could inhibit the expression of Akt and NF-kappaB p65 in the stomach and intestine of acetic acid-induced Wistar rats. This plant could be further explored for its potential as plant-based antistomach ulceration.

## 1. Introduction

Cornelius Celsus' four cardinal signs of inflammation, *rubor et tumor cum calore et dolore*, have attracted increasing attention [1, 2]. Inflammation responses play an important role in multiple diseases with a high prevalence among the population, such as hepatitis [3], lung disease [4], and Alzheimer's disease [5]. Moreover, they are also centrally related to the pathogenesis of a large number of acute and chronic diseases, i.e., colonic inflammatory response [6] and periodontitis [7].

NF-kappaB is a family of transcription factors that regulates diverse cellular activities related to inflammation and innate and adaptive immune responses [8]. Deregulation of NF-kappaB activity is implicated in the development of autoimmune diseases and cancer [9]. NF-kappaB family is composed of five structurally related members, including NF-kappaB p50, NF-kappaB p52, RelA (also named p65), RelB, and c-Rel. In most cell types, the p50/p65 heterodimer is sequestered in the cytoplasm by the inhibitor of kappaB (IkappaB), which upon stimulation is phosphorylated resulting in a polyubiquitination and degradation by the 26S proteasome. The p65 subunit then translocates into the nucleus, binds to specific NF-kappa B-sites in the enhancer regions of target genes, and regulates transcriptional activity. The activation of NF-kappaB has been observed in the inflamed tissue of the colon of patients with inflammatory bowel disease [10]. Inhibition of NF-kappaB p65 activation correlates with the ability to replace the IkappaB kinase (IKK) activities regulated by protein kinases [11–13]. Protein kinases, e.g., PI3/Akt (a downstream effector of phosphatidylinositol 3-kinase), are necessary for the activation of NF-kappaB. Phosphorylation of the p65 subunit (particularly at Ser536 located in the transactivation domain) is important in governing the strength and duration of the NF-kappaB-mediated transcriptional response [14, 15]. Thus, Akt activates IKK for NF-kappaB phosphorylation and improves the transcription ability of NF-kappaB. The Akt pathway is a signal transduction pathway that affects survival and growth in response to extracellular signals. Akt activation mediates downstream responses, including cell survival, growth, proliferation, cell migration, and angiogenesis by phosphorylation of various intracellular proteins [13].

Conventional anti-inflammatory drugs often, if not always, trigger adverse reactions in the body [14, 16, 17]; therefore, researches on plant-derived medicines have increased significantly [18]. Considering this, *Boesenbergia rotunda* (L.) Mansf. (local name *temukunci*) [19] could be proposed as a choice, since this plant has been used traditionally to reduce stomach discomforts in Asia, e.g., flatulence and diarrhea [20, 21], anti-inflammatory related to periodontitis and cancer [22, 23], anti-inflammatory [24, 25], and wound healing [26].

In this study, we investigated the mechanism of *B. rotunda* rhizome in modulating Akt and NF-kappaB p65 in the stomach and intestine of acetic acid-induced Wistar rats. Acetic acid 1% was chosen as the ulcer-inducer because the damage it causes is similar to that of human ulcers. It was reported that mucosal surface damage had occurred at 30 minutes postinjection of a single dose of acetic acid solution into the gastric mucosal layer of rats [27].

## 2. Materials and Methods

### 2.1. Plant Materials

The *B. rotunda* plants and rhizomes were purchased from the Research Institute for Spices and Medicinal Plants (BALITTRO) Manoko Lembang, West Java, Indonesia (http://balittro.litbang.pertanian.go.id/?p=993&lang=en). The plant was taxonomically identified by Djoko Kusmoro, a certified biologist at the Laboratory of Plant Taxonomy, Department of Biology, Faculty of Mathematics and Natural Sciences, Universitas Padjadjaran, Indonesia (Document No. 450/HB/10/2017).

### 2.2. Chemicals

Chemicals used were glacial acetic acid ≥99.85% (CAS Number 64-19-7, Merck) diluted with distilled water to obtain 1% v/v, ponceau S stain, Akt (44-609G, ThermoFisher Scientific, Israel), a mouse monoclonal IgG1 (kappa light chain) NF-kappaB p65 antibody (Santacruz Biotechnology Inc.), anti-mouse HRP antibody, and substrate (Li-Cor® chemiluminescence), glyceraldehyde-3-phosphate dehydrogenase (GAPDH) (Thermo Scientific AM4300, Cell Signaling Co., Ltd.), hematoxylin and eosin (H&E) (Sigma-Aldrich, Saint-Louis, USA).

### 2.3. Instruments

Instruments used were evaporator Rotavapor R215, heating bath B-491, vacuum pump V-700, distillation chiller B-741, evaporator glass-ware (Büchi, Flawil, Switzerland), chemical glass-wares (Iwaki pyrex), multimode reader Infinite M200Pro (Tecan, Grodig, Austria), digital analytical balance (Mettler-Toledo, Ohio, USA), microtubes 1.5 mL (Eppendorf, Wien, Austria), conical centrifuge tubes 50 mL (Nest, San Diego, USA), micropipette (Eppendorf, Wien, Austria), freezer −20°C (DFX40040, Marietta, OH), cold centrifuge Eppendorf AG5424 (Eppendorf, Hamburg, Jerman), pH meter (Mettler-Toledo, Ohio, USA), the cube dry bath TCDB-01 (Clever Scientific Ltd., Triton Park Brownsover Road Swift Valley Rugby Warwickshire, England), rocker shaker (Clever Scientific Ltd., Triton Park, England), GelDoc™ EZ gel documentation system (Bio-rad, International Bussiness Park, Singapore), and minigel tank (Invitrogen, Thermoscientific, Israel).

### 2.4. Extraction

The extraction procedure was carried out by adapting the method of Taweechaisupapong et al. and Woo et al.: The rhizomes were sorted, separated from dirt, washed under tap-water, thin-sliced, and sun-dried. 500 g of the dried ground rhizome of *B. rotunda* was macerated using ethanol 95% for 24 h at room temperature (25-26°C). The macerate was filtered and the residue was remacerated for 2 × 24 h [28, 29]. The macerates were collected and filtered, and the solvent was rotary-evaporated at 45°C. The viscous *B. rotunda* rhizome ethanol extract (BREE) (yield 17.24% w/w) was ready for further use.

### 2.5. Animals

Forty-eight Wistar albino rats, aged 6–8 weeks, weighed 210–240 g, were bred in the Animal Facility of PT. Biofarma, Kolonel Masturi road, Kav. 10, Kertawangi, Cisarua, West Java, 40551, Indonesia (http://maps.app.goo.gl/v7oXn3Dx3Xr633jp9). The animals were strain identified at the Laboratory of Animal Biosystematic and Plant Taxonomy, Department of Biology, Faculty of Mathematics and Natural Sciences, Universitas Padjadjaran, Indonesia (Document No. 197/HB/10/2018). Rats were kept at 24°C under a 12-hour light, 12-hour dark cycle (light was turned on from 6 am to 6 pm), 55% relative humidity, with food and water *ad libitum* for 1 week and 18 hours in the Animal Laboratory, Physiology Division, Faculty of Medicine, Universitas Padjadjaran. Animal handling, maintenance, and euthanasia procedures were performed as approved by the Ethics Committee, Faculty of Medicine, Universitas Padjadjaran, Indonesia (Document No. 1458/UN6.KEP/EC/2018 for the anti-inflammatory study and No. 1236/UN6.KEP/EC/2019 for the acute toxicity study).

### 2.6. Anti-Inflammatory Studies of the Ethanol Extract of *B. rotunda* Rhizome

Eighteen Wistar male rats, 8–10 weeks, weighed 210–240 g were randomly divided into six groups (*n* = 3): normal control (was treated with water only), positive control (was inflammation-induced and treated with quercetin 20 mg/kg BW orally by using oral gavage feeding) [30], negative control (was inflammation-induced and treated with Arabic gum suspension 2%), and 3 treatment groups (were given BREE dose of 250 mg/kg BW, 500 mg/kg BW, and 1000 mg/kg BW, all diluted in distilled water, respectively. The administration was done by using oral gavage feeding) [31, 32]. 0.1 mL of acetic acid 1% in distilled water (v/v) was injected intraperitoneally as the inflammation inducer by following the procedure of Kolgazi et al. [33] and Ozturk et al. [34] with a few modifications. The acetic acid induction was injected 30 minutes before the treatment. The rats were observed for 18 hours and the total body of each rat was weighed before the sacrifice. The rats were sacrificed using isoflurane 2% inhalation. The stomach and intestine of each rat were removed, weighed, and were rapidly frozen in liquid nitrogen and stored at −80°C for further use. The ratio of the organ per body of the rats was calculated.

### 2.7. Histopathology Analysis: Infiltration of Inflammatory Cells

The stomach and intestine of the rats were fixed in 10% v/v buffered formalin (contained 0.01 M phosphate buffer saline at pH 7.2) and were subsequently sliced to 5 *μ*m thickness, embedded in paraffin, and were H&E stained to evaluate the infiltration of inflammatory cells as described by Cantarella et al. [35] and Cantarella et al. [36]. A light microscope was used for the histopathological assessment by a total magnification of 400x and 1000x.

### 2.8. Western Blot Analysis

The dissected stomach and intestine of the rats were weighed, homogenized in lysis buffer (containing 10 mM Tris-HCl pH 7.8, 150 mM NaCl, 1 mM EDTA, 1% Nonidet P-40, and protease inhibitors). The protein samples were heat-denatured at 96°C for 5 minutes. Samples (12 *μ*g/lane) were separated by SDS-PAGE and were then transferred to a nitrocellulose membrane (GE Healthcare) for 1 hour at room temperature and blocked overnight at 4°C in Tris-buffered saline with 0.1% Tween® 20. Immunoblotting was performed using a mouse monoclonal IgG1 (kappa light chain) NF-kappaB p65 antibody (Santacruz Biotechnology Inc., diluted 1 : 100), Akt (ThermoFisher Scientific; diluted 1 : 10000 in phosphate-buffered saline 0.1% Tween® 20), and GAPDH (Santacruz Biotechnology Inc.; diluted 1 : 1000). The signals were developed using enhanced chemiluminescence reagent (GE Healthcare) and the band intensities were determined using ImageJ software (https://imagej.nih.gov/ij/). Blots were stripped and were reprobed using an anti-GAPDH mouse monoclonal antibody as the internal control to monitor the level of protein.

### 2.9. Acute Toxicity Study

The acute toxicity study (21 days) was conducted by following the Regulation of Indonesia National Agency of Drug and Food Control No. 7/2014 about In Vivo Nonclinical Toxicity Study using thirty Wistar male rats, 8–10 weeks, weighing 210–240 g. The rats were randomly divided into six groups (*n* = 5): normal control (treated with water only), and 5 treatment groups of BREE (250 mg/kg BW; 500 mg/kg BW; 1000 mg/kg BW; 2000 mg/kg BW; 4000 mg/kg BW) which were given by single administration. The BW of the rats was weighed and their physical changes of toxic effects (color changes of skin and eyes, mucous secretion, urination, defecation, strange behavior, etc.) were observed daily. On day 21^st^ the rats were sacrificed and their main organs (heart, kidney, liver, testis) were examined.

### 2.10. Statistical Analysis

SPSS 17.0 for Windows was employed to analyze the data. Significant differences between groups were analyzed using the One-Way ANOVA and post hoc Bonferroni test. All data are presented as the mean ± SEM, and the *p*-value <0.05 indicates a significant result.

## 3. Results

The solvent selection is crucial for plant extraction. Ethanol is a polar universal solvent that can extract almost all secondary metabolites in the plants [37]. The presence of flavonol quercetin in the ethanol extract of *B. rotunda* rhizome has been confirmed by employing the liquid chromatography-mass spectrometric method [38]. In this work, Arabic gum was used as the suspending agent. This gum, obtained from the dried exudate of *Acacia senegal* branches, consists of water-soluble dietary fibers. Arabic gum is commonly used to increase the digestibility of plant extracts in animal studies [39].

### 3.1. The Ratio of Organ per Body Weight

**Figure 1:**
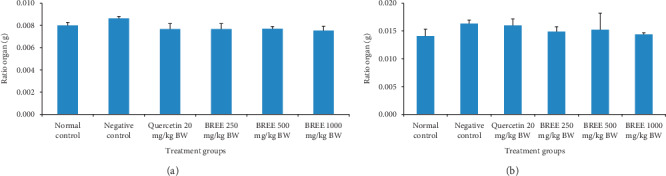
Ratio of (a) stomach/BW and (b) intestine/BW of the rats. The error bars represent the standard error of measurements (*n* = 3).

Figures 1(a) and 1(b) show the influence of BREE on the ratio of stomach/BW and intestine/BW, respectively, of the inflammation-induced rats. No significant difference in the ratio of organ/BW was observed. However, the negative control group indicated a higher ratio compared to that of other groups.

### 3.2. Histopathology Analysis: Infiltration of Inflammatory Cells

**Figure 2:**
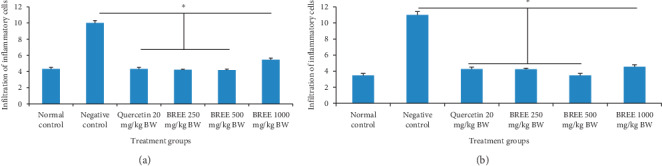
Infiltration of inflammatory cells in the stomach (a) and intestine (b) of acetic acid-induced rats. The error bars represent the standard error of measurements (*n* = 3). ^*∗*^*p* < 0.05 means significantly different compared with the negative control.

High infiltration of inflammatory cells was observed in the negative control group. BREE could reduce the infiltration of inflammatory cells in both the stomach (Figure 2(a)) and the intestine (Figure 2(b)) of acetic acid-induced rats.

**Figure 3:**
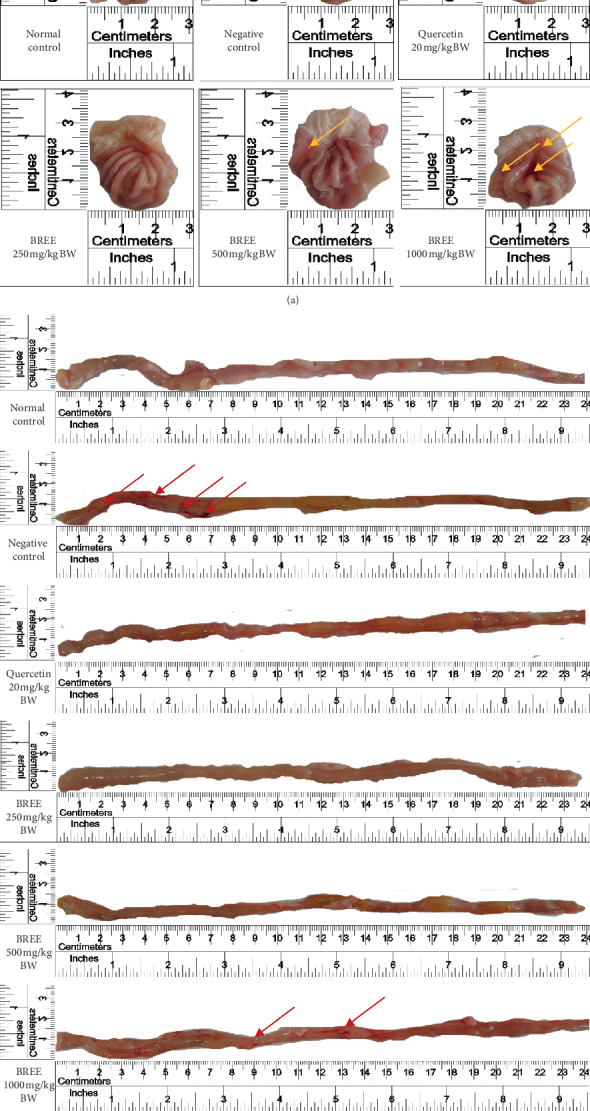
The macroscopic examination of the stomach (a) and intestine (b) of the rats. Ulceration spots were observed on the stomach (indicated by yellow arrows) of the negative control rats and the intestine (indicated by red arrows) of the negative control and BREE dose 1000 mg/kg BW rats.

The normal size of the rat's stomach is 2.5 × 2.5 cm^2^. An increase of the size is observed in all acetic acid-induced groups, with the exception in the group treated with the highest dose of BREE (Figure 3). Furthermore, the organ examination (Figure 3) indicated that ulceration spots were observed on the stomach (yellow arrows) and intestine (red arrows) of the negative control groups. Surprisingly, the highest dose of BREE (1000 mg/kg BW) also elicits ulceration in both the stomach and intestine.

**Figure 4:**
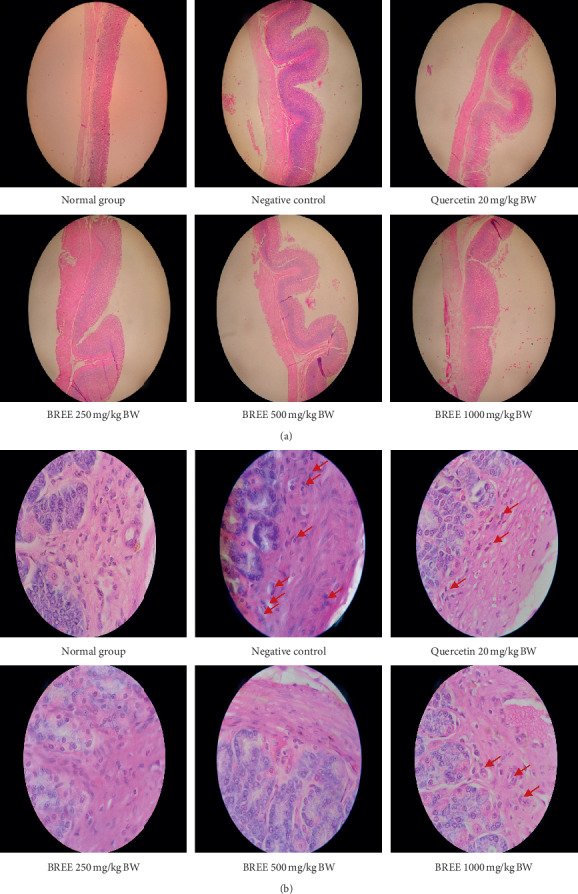
Microscopic histopathology examination of the stomach of the rats. Objective lens 4x (a) and 100x (b). Red arrows indicate granulocyte neutrophils.

**Figure 5:**
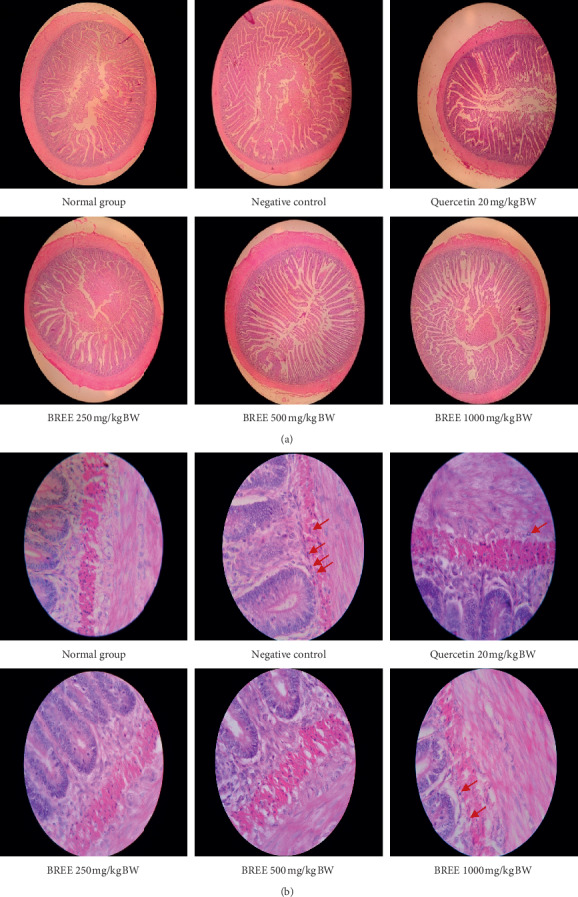
Microscopic histopathology examination of the intestine of the rats. Objective lens 4x (a) and 100x (b). Red arrows indicate granulocyte neutrophils.

The histopathology of the stomach is presented in Figure 4(a) (H&E stain, objective lense 4x) and Figure 4(b) (H&E stain, objective lense 100x). Histopathology of the intestine is presented in Figure 5(a) (H&E stain, objective lense 4x) and Figure 5(b) (H&E stain, objective lense 100x). An active inflammation indicated by the infiltration of granulocyte neutrophils was observed in the negative control (ulceration index = 1.5 both in the stomach and intestine) as well as in the rats treated with quercetin (ulceration index = 0.33 both in the stomach and intestine) and the highest dose of BREE (ulceration index = 1.0 in the stomach and intestine).

### 3.3. Western Blot Analysis

**Figure 6:**
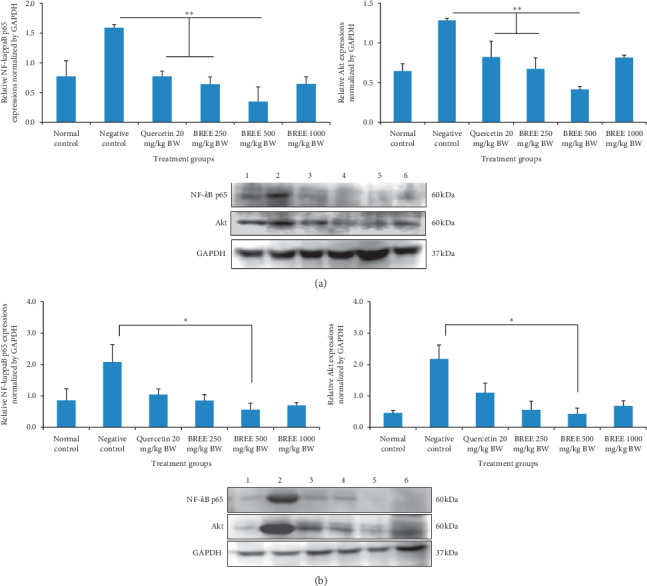
The bands of NF-kappaB p65 (60 kDa), Akt (60 kDa), and GAPDH (37 kDa) in the stomach (a) and the intestine (b) of the Wistar rats by Western blot analysis. ^*∗*^*p* < 0.05 and ^*∗∗*^*p* < 0.01 means significantly different compared with the normal control (calculated by One-Way ANOVA followed with post hoc Bonferroni test). 1 = Normal, 2 = Negative Control, 3 = Quercetin 20 mg/kg BW, 4 = BREE 250 mg/kg BW, 5 = BREE 500 mg/kg BW, 6 = BREE 1000 mg/kg BW.

Figures 6(a) and 6(b) show the bands of NF-kappaB p65 (60 kDa), Akt (60 kDa), and GAPDH (37 kDa) in the stomach and the intestine of the Wistar rats, respectively. The relative expressions of NF-kappaB p65 (Figure 6(a) middle part) and Akt (Figure 6(a) lower part) normalized by GAPDH in the stomach at a dose of 250 mg/kg BW are 0.64 ± 0.09 and 0.68 ± 0.14, respectively, whereas a dose of 500 mg/kg BW is 0.35 ± 0.24 and 0.41 ± 0.04, respectively. This inhibitory activity on Akt expression and NF-kappaB p65 is better than that of quercetin (the positive control). However, an increase of this expression was observed at dose 1000 mg/kg BW of BREE.

Similarly, the relative expressions of NF-kappaB p65 (Figure 6(b) middle part) and Akt (Figure 6(b) lower part) normalized by GAPDH in the intestine at a dose of 250 mg/kg BW are 1.05 ± 0.30 and 0.55 ± 0.27, respectively, whereas a dose of 500 mg/kg BW is 0.56 ± 0.21 and 0.43 ± 0.19, respectively. This inhibitory activity on Akt expression and NF-kappaB p65 is better than that of quercetin (the positive control). However, an increase of this expression was observed at a dose of 1000 mg/kg BW of BREE.

### 3.4. Acute Toxicity Study (21 Days)

**Figure 7:**
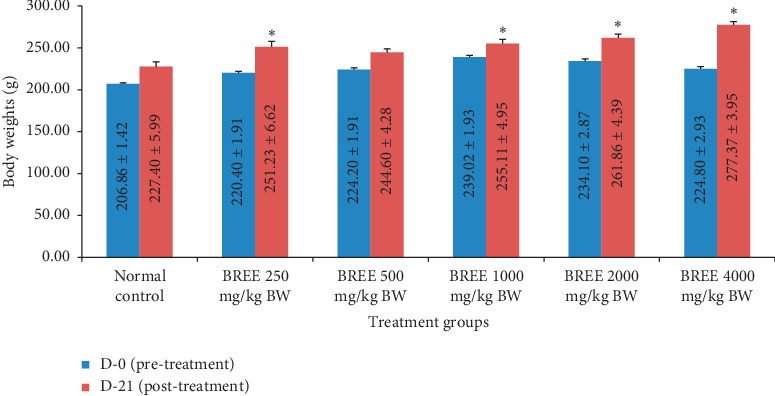
The BW of the rats at D-0 (pre-) and D-21 (post-) BREE treatment. ^*∗*^*p* < 0.05 means significantly different compared with the normal control.

**Figure 8:**
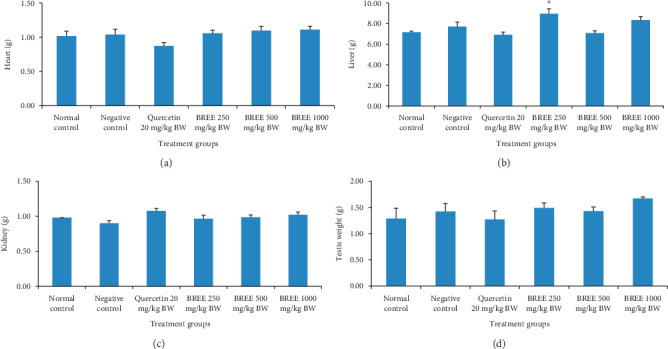
The ratio of organ/BW on day 21^st^: heart (a); liver (b); kidney (c); testis (d). ^*∗*^indicated *p* < 0.01 (*p*=0.038).

During 21 consecutive days of observation after a single oral BREE-administration, the rats did not show any sign of toxicity nor death which indicates that the LD_50_ of BREE is greater than 4000 mg/kg BW. The general appearance, grooming, posture, behavior of the rats were normal. The BW of the rats was significantly different between D-0 and D-21 of the study (Figure 7), except for the normal control group and rats treated with BREE dose 500 mg/kg BW. The ratio of organ/BW on day 21^st^ is presented in Figure 8.

## 4. Discussion

More than 370 million people representing 5000 distinct groups had received global recognition, namely, the United Nations Declaration on the Rights of Indigenous Peoples (UNDRIP), in which it affirmed the rights of indigenous peoples to their traditional medicines and health practices [40]. A family survey in Indonesia, which was carried out during 2014-2015, reported that a high prevalence of indigenous medicine was used in children and several social factors and poor health status of its use were identified [41].

The rhizome of *Boesenbergia rotunda* (local name *temukunci*) has been used traditionally to reduce stomach discomforts [20, 21]. This plant's anti-inflammatory activity has also been reported [22–26]. Quercetin, a flavonol, has been reported to be contained in the rhizome of this plant [38]. Our work indicated that the ethanol extract of *B. rotunda* rhizome (BREE) obtained from Lembang, West Java, Indonesia, did not influence the ratio of stomach/BW and intestine/BW of the inflammation-induced rats.

Lower doses of BREE could significantly (*p* < 0.01) reduce the infiltration of inflammatory cells (indicated by the absence of the granulocyte neutrophils) in both the stomach and the intestine of acetic acid-induced rats. The infiltration of granulocyte neutrophils was observed in the stomach and intestine of rats of the negative control group, the positive control group, and rats treated with dose 1000 mg/kg BW of BREE (indicated by red arrows in Figures 4(b) and 5(b)). This infiltration of neutrophils indicated an occurrence of inflammation in both organs. Quercetin used in our study (20 mg/kg BW) has caused a slight inflammation in the rat's stomach and intestine. This result is similar to that of Tang et al.; a higher concentration of quercetin (15.0 *μ*g/ml) exposed to RAW264.7 cells for 24 hours that was reported could significantly decrease the cell viability [42].

The granulocyte neutrophils are produced in the bone marrow, and upon activation by various cytokines, they transit via the vascular endothelium during the recruitment process and enter the inflammatory site. Subsequently, they become fully activated as characterized by the release of their granule proteins [43]. These granulocyte neutrophils are also called polymorphonuclear neutrophils or polymorphs [44].

In the Western blot experiment, it was confirmed that the NF-kappaB signaling pathway was activated in the acetic acid-induced rats and yet this pathway was blocked by lower doses of BREE. The doses 250 mg/kg BW and 500 mg/kg BW of BREE could inhibit the expression of Akt and NF-kappaB p65 in the stomach and intestine of the acetic acid-induced rats better than that of quercetin (the positive control). Akt plays an important role in the activation of NF-kappaB. Thus, the inhibition of Akt expression indirectly inactivates the NF-kappaB and reduces the expression of the p65 subunit. However, an increase of NF-kappaB p65 and Akt expression was observed in rats treated with the highest dose (1000 mg/kg BW) of BREE (Figure 6). This result, nonetheless, is in agreement with the macroscopic examination (Figure 3(b)). Figure 3(b) revealed that the highest dose of BREE (1000 mg/kg BW) elicits ulceration in the intestine.

A higher concentration of plant extract could induce abnormality in the animal. A previous study on the anti-inflammatory activity of *Boswellia serrata* extract reported that the highest concentration of the extract (100 *μ*g dry extract/mL) was found to be cytotoxic and decreased the viability of porcine aortic endothelial cells, while lower concentrations did not affect the cell viability [45].

**Figure 9:**
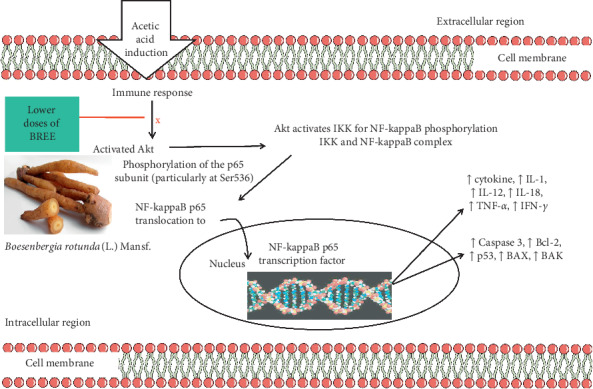
Proposed anti-inflammatory mechanism of BREE in acetic acid-induced Wistar rats. Lower doses of BREE (250 mg/kg BW and 500 mg/kg BW) inhibit the activation of Akt thus resulting in the decrease of NF-kappaB p65 in the nucleus.

Taken together, BREE reduces stomach ulceration in rats by inhibiting the activation of Akt; thus the phosphorylation of NF-kappaB p65 at Ser536 is blocked and NF-kappaB p65 could not translocate to the nucleus (Figure 9).

A recent study on the TLR4-dependent activation of NF-kappaB in bone marrow-derived and peritoneal primary macrophages indicated that the total amount of cytoplasmic and nucleus NF-kappaB remained unchanged. The NF-kappaB level in the nucleus reached its maximum at 10 minutes after lipopolysaccharide induction, then it gradually decreased to a 50% level at 100 minutes. Nonetheless, the kinetics of NF-kappaB translocation into the nucleus was reported slightly slower than its phosphorylation rate [46].

The main objective of evaluating the safety of any medicinal plant is to identify the nature and significance of toxicity and to establish the exposure level at which this toxicity is observed [47]. Our 21 days of toxicity study indicated that after a single oral BREE treatment, the Wistar rats did not show any sign of toxicity nor death, which indicates that the LD_50_ of BREE is greater than 4000 mg/kg BW.

The acute toxicity of the *B. rotunda* extract had been evaluated in normal healthy Wistar rats for 72 hours [32] and in Sprague Dawley rats for 14 days [48]. The rats were subjected to a single dose of 2000 mg/kg BW [32] and 5000 mg/kg BW [48] of the rhizome extract preparation, respectively. It was reported that all the rats in this test remained alive and did not manifest mortality or any visible signs of toxicity [32, 48]. The clinical biochemistry measurements of the serum reflected a normal function of the kidney (sodium, potassium, and chloride) and the liver (total bilirubin, alkaline phosphatase, alanine aminotransferase, aspartate aminotransferase, and gamma-glutamyl transferase) [48].

## 5. Conclusions

The peptic-ulceration induced by acetic acid could be inhibited by the ethanol extract of *Boesenbergia rotunda* (L.) Mansf. rhizome. Specifically, the rhizome of this plant inhibits the activation of Akt thus resulting in a decrease of NF-kappaB p65 expression in the stomach and intestine of acetic acid-induced Wistar rats. Flavonoids contained in the rhizome extract might play a key role in this anti-inflammatory activity. However, this plant still needs further exploration for its potential as plant-based antistomach ulceration.

## Data Availability

The data used to support the findings of this study are included in the article.
